# Supplementary material for: Garcienone, a Novel Compound Involved in Allelopathic Activity of Garcinia Xanthochymus Hook
Source: Plants (Basel). 2019 Aug 24;8(9):301. doi: 10.3390/plants8090301 (PMC6784076; doi:10.3390/plants8090301)
Supplement: Supplementary file 1 [file plants-08-00301-s001.pdf]

Fig. S1. ^{1}H NMR (400 MHz, $CDCl_3$)

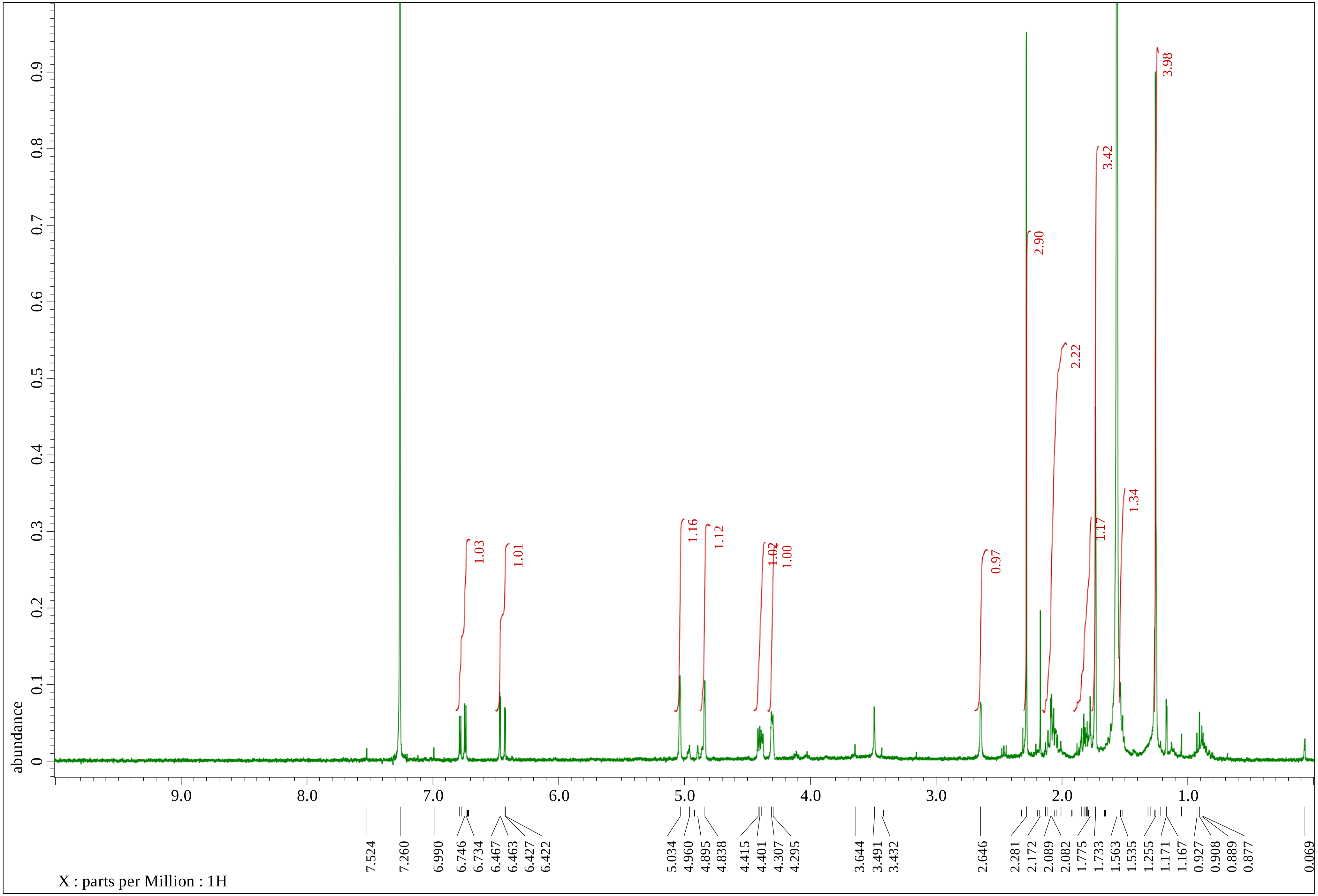

Fig. S2. ^{13}C NMR (100 MHz, $CDCl_3$)

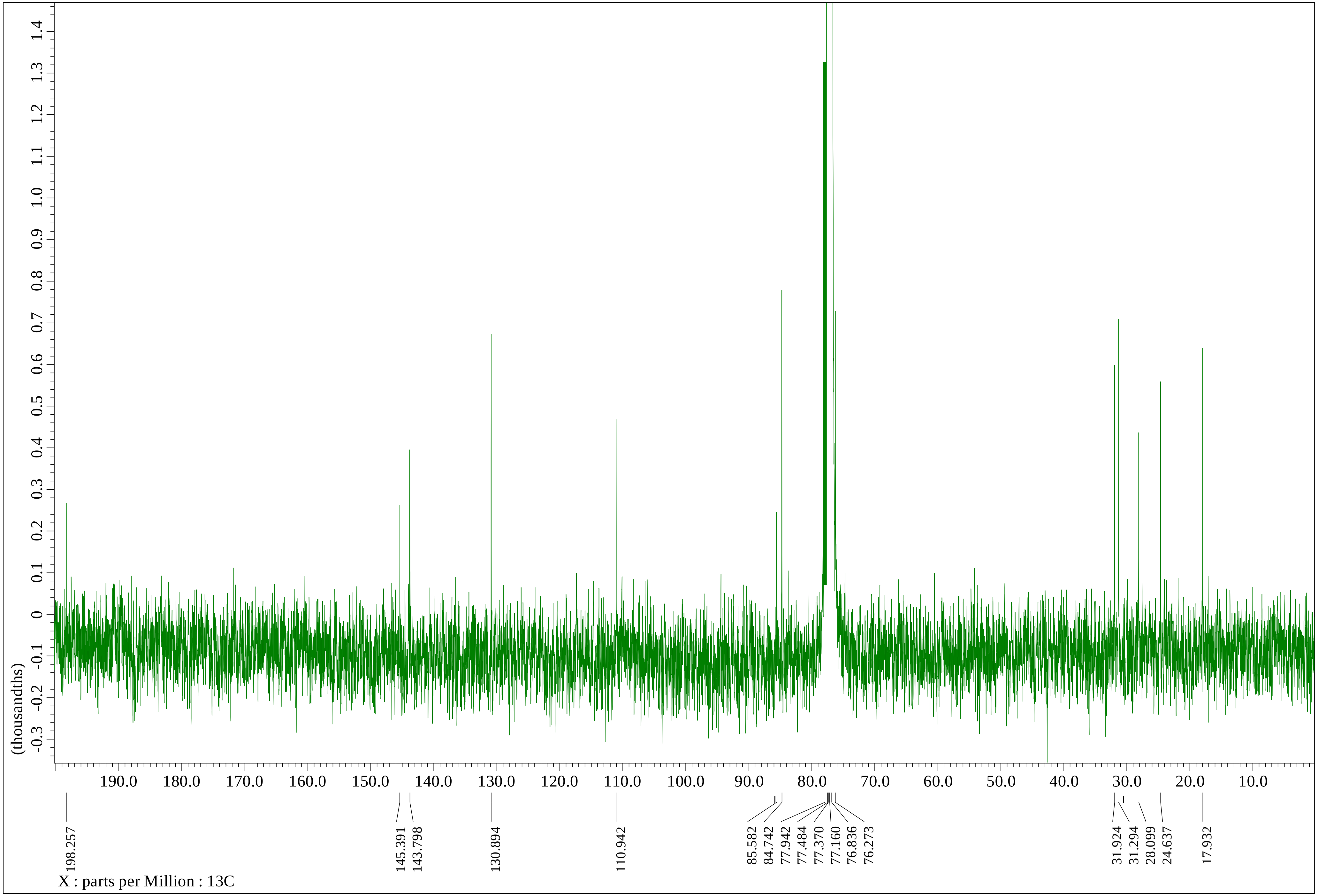

Fig. S3. COSY (400 MHz, CDCl3)

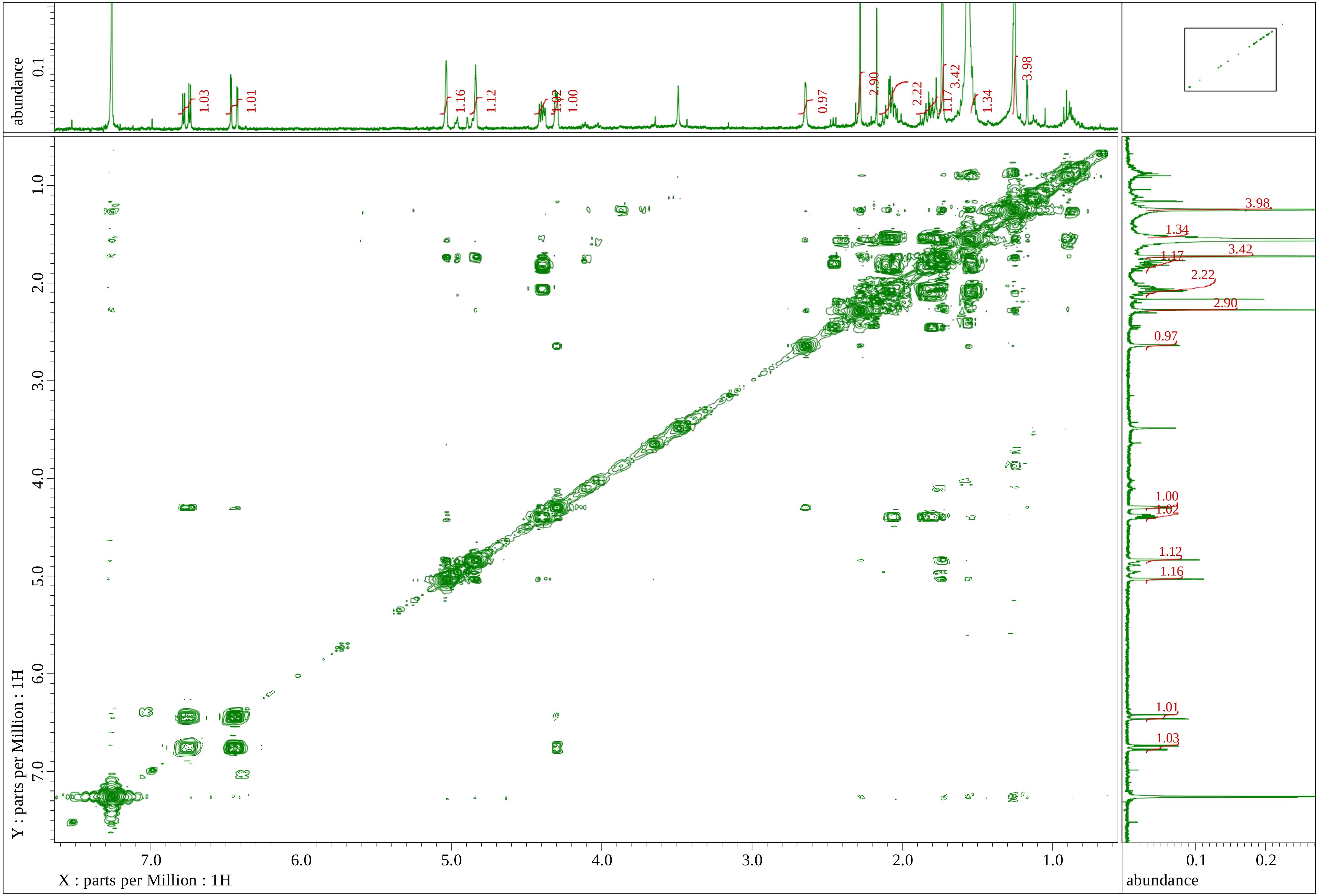

Fig.S4. HMBC (400 MHz,

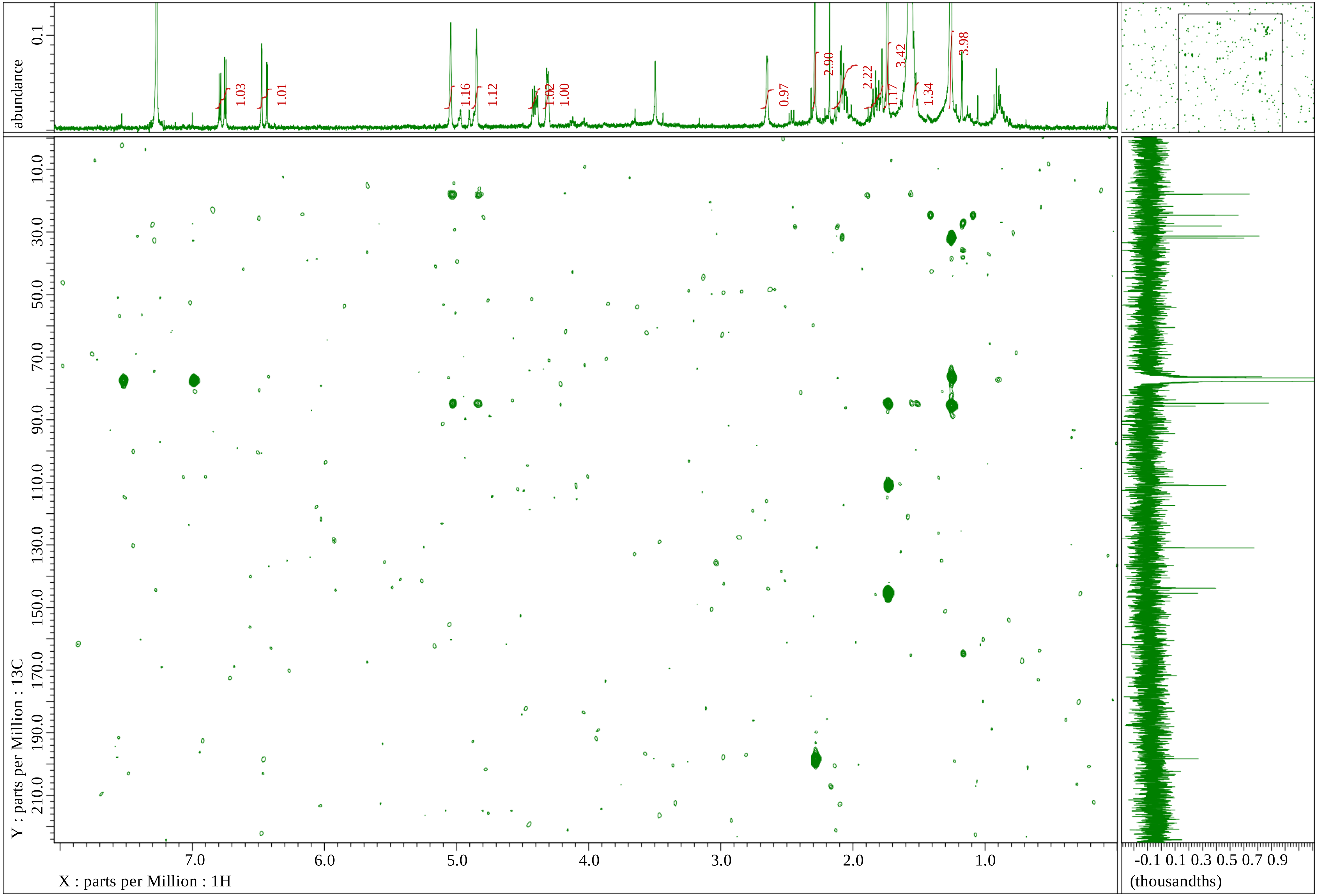

Fig. S5. HMQC (400

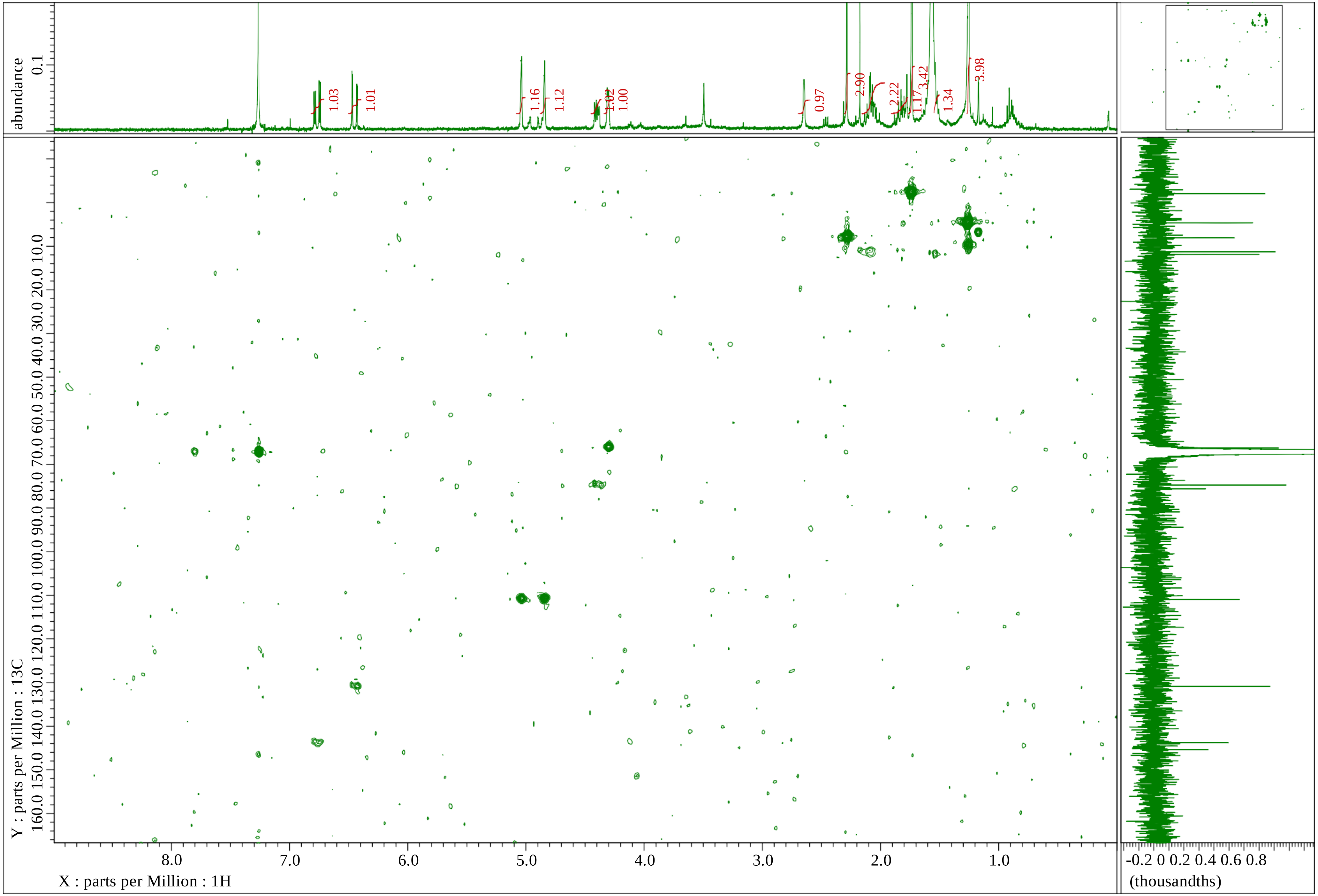

Fig. S6. NOESY (400 MHz, CDCl3)

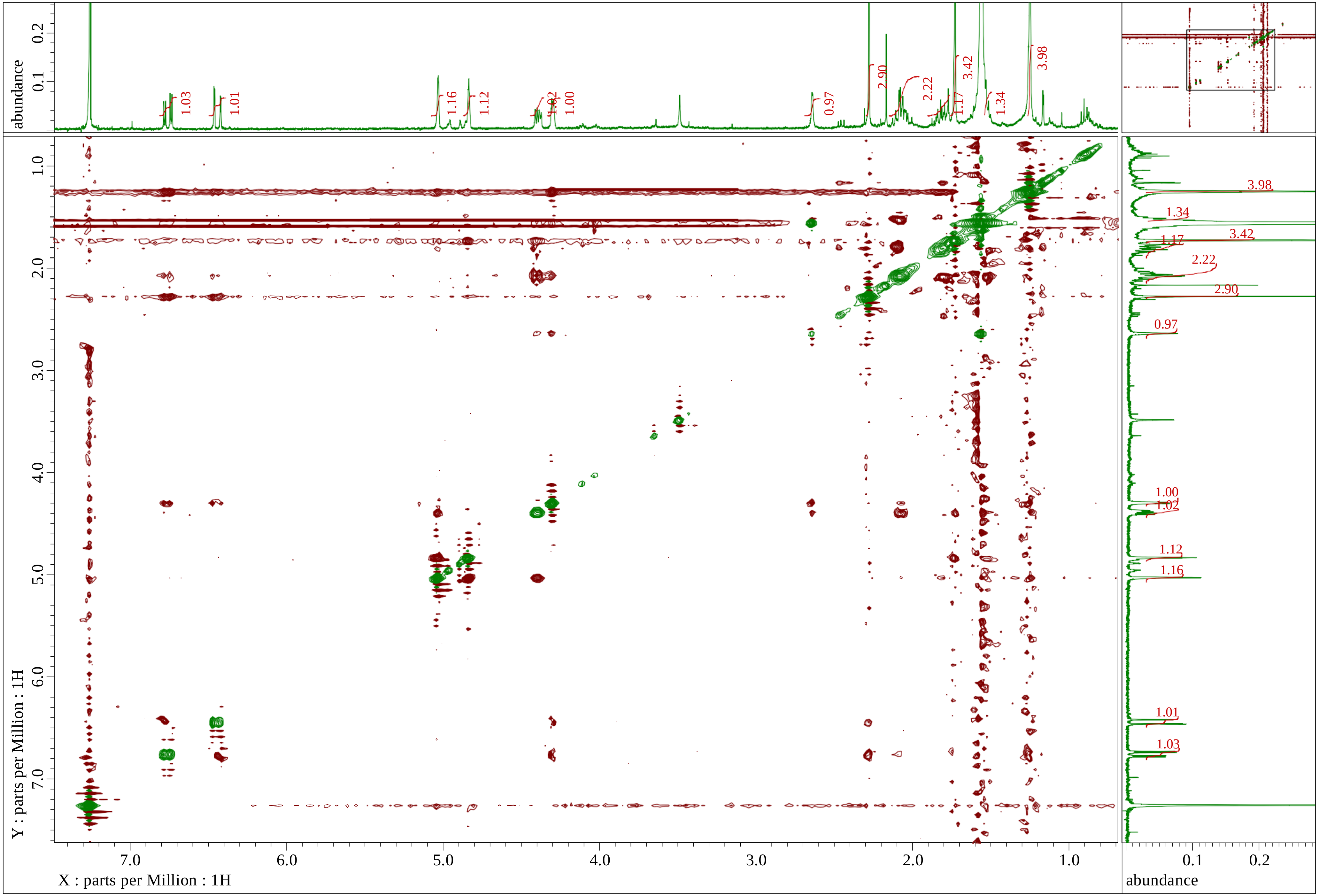

Fig. S7. 1D NOE (400 MHz, CDCl3)

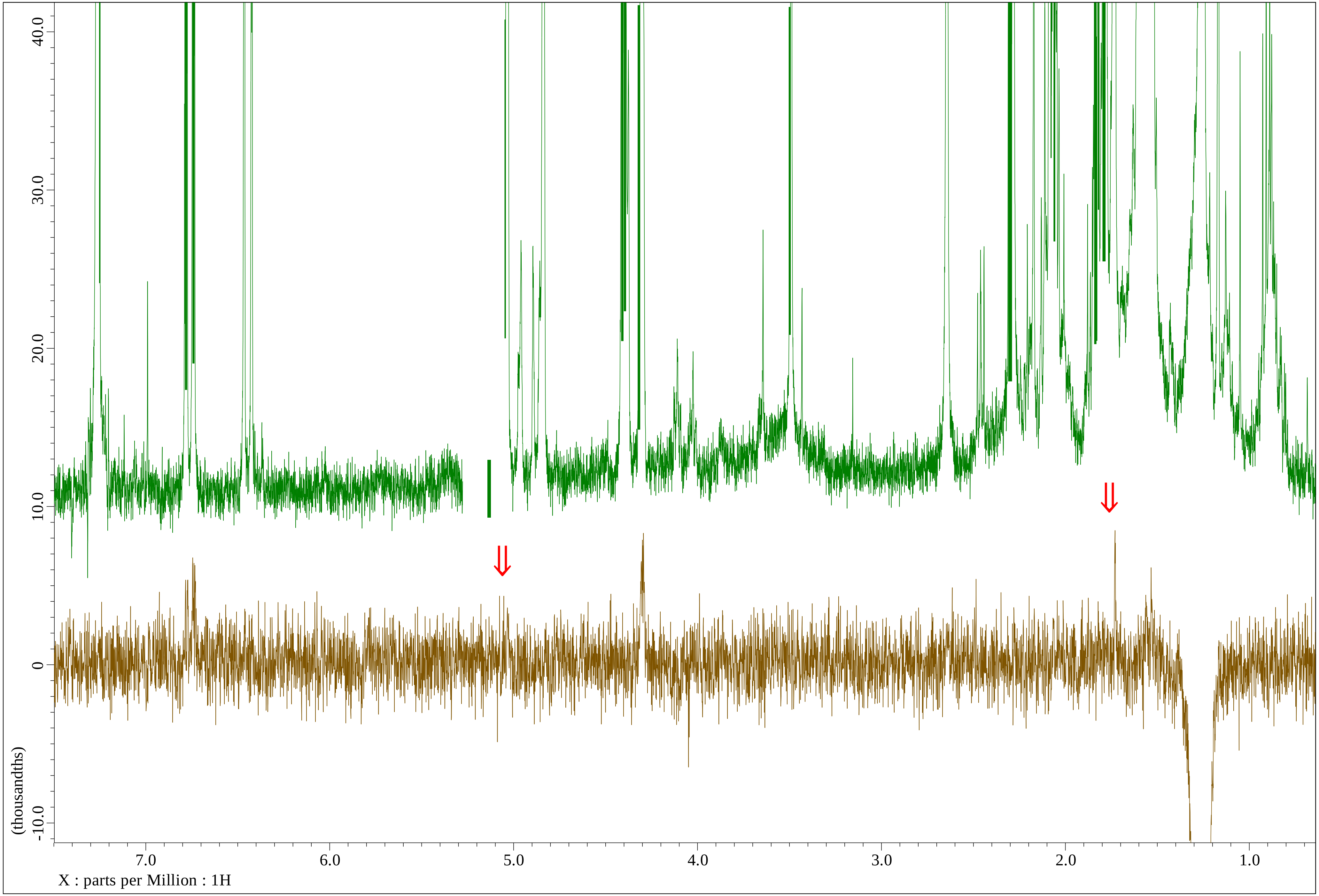

NMR chemical shifts calculation and DP4+ analyses.

Table S 1. Results of DP4+ analysis of garcienone.

Functional		Solvent?		Basis Set	Type of Data	
mPW1PW91		PCM		6-31+G(d,p)	Shielding Tensors	
		DP4+	0.00%	100.00%	0.00%	0.00%	-
Nuclei	sp2?	Experimenta	Isomer 1	Isomer 2	Isomer 3	Isomer 4	Isomer 5
C		31.9	160.8	163.8	161.7	160.0	
C		85.6	107.8	107.9	107.6	108.0	
C		84.7	113.8	111.7	114.1	113.3	
C		31.3	165.7	161.0	158.7	163.5	
C	x	145.4	48.6	48.6	48.8	49.0	
C		24.6	173.3	171.4	170.8	172.4	
C		76.3	118.8	118.9	116.6	116.2	
C	x	143.8	51.0	51.4	51.1	50.0	
C	x	130.9	72.6	72.7	72.1	70.7	
C	x	198.3	2.2	2.2	2.3	1.5	
C		28.1	164.1	164.2	164.0	165.4	
C		17.9	173.80	174.63	174.49	173.88	
C	x	110.9	90.51	91.28	91.51	89.62	
H		1.53	29.73	30.22	29.56	29.43	
H		2.09	29.54	29.48	30.01	29.84	
H		4.39	27.01	27.05	26.98	26.95	
H		2.06	29.46	29.40	29.47	29.46	
H		1.82	29.27	29.87	29.90	29.53	
H		1.26	30.55	30.27	30.26	30.48	
H		4.3	27.20	27.01	27.08	27.42	
H	x	6.76	24.11	24.07	24.06	24.13	
H	x	6.45	24.53	24.55	24.54	24.84	
H		2.28	29.31	29.30	29.30	29.29	
H		1.73	29.64	29.82	29.80	29.67	
H	x	4.84	26.42	26.48	26.42	26.39	
H	x	5.03	26.3890113	26.0085771	26.1165759	26.2718765	

Isomer 1: 5*S*6*S*9*S*-**1**,

Isomer 2: 5*R*6*S*9*S*-**1**,

Isomer 3: 5*S*6*R*9*S*-**1**,

Isomer 4: 5*R*6*R*9*S*-**1**

The data of isomers 1-4 are shown as shielding tensors. These values can be converted to chemical shifts, and we show the corresponding chemical shifts in Table S 2 in the next page.

Table S 2. Experimental and calculated chemical shift values of garcienone and its possible isomers, 5*S*6*S*9*S*, 5*R*6*S*9*S*, 5*S*6*R*9*S* and 5*R*6*R*9*S*

	Experimental	5S6S9S		5R6S9S		5S6R9S		5R6R9S	
	Chemical Shifts	Shielding Tensor	Chemical Shifts	Shielding Tensor	Chemical Shifts	Shielding Tensor	Chemical Shifts	Shielding Tensor	Chemical Shifts
C	31.9	160.8	35.8	163.8	32.7	161.7	34.9	160.0	36.6
C	85.6	107.8	88.8	107.9	88.7	107.6	89.0	108.0	88.6
C	84.7	113.8	82.7	111.7	84.9	114.1	82.5	113.3	83.3
C	31.3	165.7	30.9	161.0	35.6	158.7	37.9	163.5	33.1
C	145.4	48.6	147.9	48.6	148.0	48.8	147.7	49.0	147.6
C	24.6	173.3	23.2	171.4	25.2	170.8	25.8	172.4	24.2
C	76.3	118.8	77.8	118.9	77.7	116.6	79.9	116.2	80.4
C	143.8	51.0	145.5	51.4	145.2	51.1	145.4	50.0	146.5
C	130.9	72.6	124.0	72.7	123.9	72.1	124.5	70.7	125.9
C	198.3	2.2	194.4	2.2	194.4	2.3	194.3	1.5	195.1
C	28.1	164.1	32.5	164.2	32.4	164.0	32.6	165.4	31.2
C	17.9	173.8	22.8	174.6	21.9	174.5	22.1	173.9	22.7
C	110.9	90.5	106.1	91.3	105.3	91.5	105.1	89.6	107.0
H	1.53	29.73	1.83	30.22	1.34	29.56	2.00	29.43	2.13
H	2.09	29.54	2.02	29.48	2.08	30.01	1.55	29.84	1.72
H	4.39	27.01	4.55	27.05	4.51	26.98	4.58	26.95	4.61
H	2.06	29.46	2.10	29.40	2.16	29.47	2.09	29.46	2.10
H	1.82	29.27	2.29	29.87	1.69	29.90	1.66	29.53	2.03
H	1.26	30.55	1.01	30.27	1.29	30.26	1.30	30.48	1.08
H	4.30	27.20	4.36	27.01	4.55	27.08	4.48	27.42	4.14
H	6.76	24.11	7.44	24.07	7.49	24.06	7.50	24.13	7.43
H	6.45	24.53	7.03	24.55	7.01	24.54	7.02	24.84	6.72
H	2.28	29.31	2.25	29.30	2.26	29.30	2.26	29.29	2.27
H	1.73	29.64	1.92	29.82	1.73	29.80	1.76	29.67	1.89
H	4.84	26.42	5.14	26.48	5.08	26.42	5.13	26.39	5.17
H	5.03	26.39	5.17	26.01	5.55	26.12	5.44	26.27	5.29

Table S 3. Energy and Boltzmann distribution of the energy-minimized conformers of 5*S*6*S*9*S* isomer optimized at the B3LYP/6-31G* level.

The conformers within 2 kcal/mol of the global minimum are shown in red. Regarding these conformers, we calculated the shielding tensors, and the values are indicated as shown below.

Conformation No.	1	2	3	4	5	6	7	8	9	10	11	12	13
Energy (A.U.)	-733.0507	-733.0542	-733.0508	-733.052	-733.0485	-733.0516	-733.0508	-733.0468	-733.0483	-733.0456	-733.0474	-733.0474	-733.0474
Energy (kcal/mol)	-459996.7	-459998.9	-459996.7	-459997.5	-459995.3	-459997.2	-459996.7	-459994.2	-459995.1	-459993.4	-459994.6	-459994.6	-459994.6
Relative Energy (kcal/mol)	2.21	0.00	2.14	1.40	3.58	1.68	2.18	4.65	3.75	5.42	4.30	4.30	4.30
Boltzmann population (%)		86.69%		8.21%		5.09%							
C		161.0466		158.7332		159.3338							
C		107.768		107.5754		107.8528							
O		230.6548		243.6164		227.9025							
C		114.2361		111.0584		111.2906							
C		166.3317		161.3817		161.2345							
C		48.5531		48.6253		49.9078							
C		173.5195		170.8736		174.455							
C		118.8055		119.9643		116.8697							
C		51.4276		47.265		50.3651							
C		72.8511		72.8505		67.2699							
C		2.2095		2.7002		0.8286							
C		163.8017		163.8272		169.4645							
O		-192.5064		-195.0541		-245.9454							
O		306.4274		308.9729		295.3184							
C		173.4722		174.4236		178.4612							
C		90.6992		91.6655		85.3605							
H		29.7325		29.778		29.6915							
H		29.5934		28.9798		29.5749							
H		27.0013		27.0656		26.9997							
H		29.4549		29.3394		29.7865							
H		29.202		29.8901		29.3776							
H		30.8561		30.3869		30.8506							
H		29.9776		30.3294		29.9604							
H		30.894		30.1829		30.4963							
H		27.1925		27.3396		27.0917							
H		24.1342		23.628		24.5601							
H		24.4996		24.6222		24.919							
H		29.1137		29.1254		29.054							
H		29.713		29.7111		29.7279							
H		29.098		29.1089		29.0699							
H		28.0778		29.1756		28.353							
H		29.1744		29.9274		29.6577							
H		29.8474		29.9076		29.4088							
H		29.8363		29.7275		29.9305							
H		26.4055		26.5471		26.4077							
H		26.421		26.1126		26.2903							

Table S 4. Energy and Boltzmann distribution of the energy-minimized conformers of 5*R*6*S*9*S* isomer optimized at the B3LYP/6-31G* level.

The conformers within 2 kcal/mol of the global minimum are shown in red. Regarding these conformers, we calculated the shielding tensors, and the values are indicated as shown below.

Conformation No.	1	2	3	4	5	6	7	8	9	10	11	12	13	14	15	16
Energy (A.U.)	-733.0507	-733.0542	-733.0492	-733.051	-733.0488	-733.0518	-733.0492	-733.0491	-733.0477	-733.0474	-733.0472	-733.0484	-733.0454	-733.048	-733.048	-733.047
Energy (kcal/mol)	-459996.7	-459998.9	-459995.7	-459996.8	-459995.4	-459997.3	-459995.7	-459995.6	-459994.8	-459994.6	-459994.4	-459995.2	-459993.3	-459995	-459995	-459994
Relative Energy (kcal/mol)	2.21	0.00	3.16	2.05	3.42	1.52	3.18	3.24	4.09	4.29	4.43	3.68	5.53	4.20	4.09	4.68
Boltzmann population (%)		92.88%				7.12%										
C		163.8631				163.6333										
C		107.925				107.7736										
O		230.6972				223.9599										
C		111.8782				108.7863										
C		160.8512				162.3709										
C		48.4904				49.6317										
C		171.3939				170.9159										
C		118.8835				118.911										
C		51.5245				50.0657										
C		73.0601				67.604										
C		2.2943				0.8131										
C		163.7983				169.4536										
O		-193.6529				-246.4165										
O		305.3491				304.7859										
C		174.3502				178.3284										
C		91.729				85.4136										
H		30.2177				30.2007										
H		29.4874				29.425										
H		27.0521				26.9706										
H		29.3684				29.8346										
H		29.8951				29.4773										
H		30.395				30.4029										
H		30.3333				30.3276										
H		30.0842				29.9997										
H		27.0118				27.0351										
H		24.0418				24.4118										
H		24.5172				24.952										
H		29.0814				29.0738										
H		29.7052				29.7255										
H		29.1213				29.0451										
H		28.334				28.3889										
H		29.9106				29.6686										
H		29.8899				29.4172										
H		29.7093				29.9243										
H		26.4883				26.3995										
H		25.9853				26.3121										

Table S 5. Energy and Boltzmann distribution of the energy-minimized conformers of 5*S*6*R*9*S* isomer optimized at the B3LYP/6-31G* level.

The conformers within 2 kcal/mol of the global minimum are shown in red. Regarding these conformers, we calculated the shielding tensors, and the values are indicated as shown below.

Conformation No.	1	2	3	4	5	6	7	8	9	10	11	12	13	14	15
Energy (A.U.)	-733.0512	-733.051	-733.0488	-733.0479	-733.0481	-733.0541	-733.0466	-733.0494	-733.051	-733.0458	-733.0512	-733.0419	-733.0457	-733.0503973	-733.0506
Energy (kcal/mol)	-459997	-459996.8	-459995.5	-459994.9	-459995	-459998.8	-459994.1	-459995.8	-459996.9	-459993.6	-459996.9	-459991.1	-459993.5	-459996.4548	-459996.6
Relative Energy (kcal/mol)	1.79	1.91	3.29	3.87	3.75	0.00	4.66	2.94	1.90	5.16	1.82	7.63	5.28	2.30	2.16
Boltzmann population (%)	4.15%	3.39%				85.07%			3.46%		3.92%				
C	159.3307	165.5527				161.6518			162.5868		160.7704				
C	108.3852	106.2025				107.6009			107.9864		107.3347				
O	239.3851	219.7864				227.9949			220.5945		231.2495				
C	111.4716	114.9555				114.2702			112.0621		114.7362				
C	161.5224	166.9422				158.2265			159.3295		158.4558				
C	49.5449	49.5986				48.7366			50.4915		48.4031				
C	170.0212	172.251				170.6929			171.2571		171.0614				
C	118.1394	120.55				116.4499			117.4659		115.0134				
C	46.4579	50.9125				51.4873			49.9512		49.6013				
C	70.4573	73.1492				72.4628			67.1035		69.1754				
C	2.1155	2.2046				2.377			0.6663		2.5776				
C	164.1051	163.7463				163.7349			169.4448		163.8428				
O	-196.0662	-193.1907				-193.6524			-247.1466		-203.1471				
O	305.5255	302.2966				296.1176			298.5164		293.4637				
C	177.699	172.4386				174.2469			178.6463		174.3532				
C	85.1153	88.7645				92.1584			85.333		92.0754				
H	29.3176	29.4775				29.5845			29.5402		29.3237				
H	29.6252	30.3833				30.0142			30.0604		29.9243				
H	27.0684	26.9759				26.9801			26.9632		26.9888				
H	29.8814	29.3196				29.4381			29.8832		29.3751				
H	29.74	29.4128				29.9456			29.6129		29.8217				
H	29.4928	30.3993				30.248			30.3432		30.4299				
H	30.4691	30.5145				30.311			30.4323		30.5359				
H	30.455	30.0081				30.1925			30.066		30.4099				
H	27.4155	27.127				27.0512			27.0237		27.3936				
H	23.7859	24.0265				24.0557			24.4027		24.1182				
H	24.4587	24.5596				24.5104			24.9177		24.8742				
H	29.0578	29.1094				29.0918			29.0579		29.199				
H	29.1404	29.1475				29.1162			29.0355		29.1137				
H	29.6842	29.7224				29.6985			29.7111		29.6873				
H	29.2631	28.8368				28.6593			28.4016		28.9385				
H	30.0642	29.3834				29.8709			29.7056		29.8657				
H	29.8659	29.7921				29.8387			29.5425		29.814				
H	30.1356	29.773				29.6881			29.8187		29.6906				
H	26.5463	26.2612				26.427			26.3725		26.4256				
H	26.3625	26.3394				26.0902			26.2805		26.0911				

Table S 6. Energy and Boltzmann distribution of the energy-minimized conformers of 5*R*6*R*9*S* isomer optimized at the B3LYP/6-31G* level.

The conformers within 2 kcal/mol of the global minimum are shown in red. Regarding these conformers, we calculated the shielding tensors, and the values are indicated as shown below.

Conformation No.	1	2	3	4	5	6	7	8	9	10	11	12	13
Energy (A.U.)	-733.0509	-733.049	-733.048	-733.049	-733.0505	-733.0485	-733.0435	-733.049	-733.0398	-733.0484	-733.0483	-733.0438	-733.0458
Energy (kcal/mol)	-459996.8	-459995.6	-459994.9	-459995.6	-459996.5	-459995.3	-459992.1	-459995.6	-459989.8	-459995.2	-459995.2	-459992.3	-459993.5
Relative Energy (kcal/mol)	0.00	1.19	1.87	1.19	0.29	1.51	4.66	1.19	6.98	1.60	1.64	4.48	3.25
Boltzmann population (%)	44.07%	5.90%	1.87%	5.93%	27.09%	3.46%		5.93%		2.98%	2.77%		
C	161.1541	160.9699	161.4248	162.0303	156.2764	161.9538		162.0335		161.7958	161.4023		
C	107.5857	106.1736	106.8566	108.9983	108.7911	107.4083		108.9974		109.42	106.7598		
O	230.1255	232.5384	228.2625	229.8141	241.0689	234.2972		229.812		235.6857	228.5243		
C	113.479	114.2667	113.188	111.1441	113.5762	114.3147		111.1481		113.863	113.3026		
C	166.0359	167.7248	166.3309	161.1318	158.6891	167.5668		161.1314		161.108	166.2701		
C	48.8149	49.5027	48.998	48.7728	49.0158	49.0847		48.7736		50.6272	48.57		
C	174.143	171.7447	174.1645	169.9846	170.854	171.7791		169.9856		169.7144	174.1249		
C	115.5057	121.4174	114.1817	117.3045	115.3976	120.3527		117.3055		117.0531	114.4634		
C	51.8023	47.0478	47.715	48.8285	48.6173	49.7014		48.832		49.5994	49.7037		
C	73.1186	72.8786	65.636	73.5299	65.094	70.4889		73.5274		73.3602	70.5903		
C	2.2398	2.7044	0.1634	1.8931	-0.3721	2.1592		1.8946		2.5952	2.5202		
C	163.8465	163.826	169.4011	163.8824	169.2367	163.9088		163.8823		163.8111	163.927		
O	-192.6867	-194.0751	-249.9889	-196.9113	-250.743	-202.7019		-196.8912		-196.7779	-204.4468		
O	294.8869	305.8721	290.3773	297.359	288.3314	290.1629		297.3655		297.1811	290.4388		
C	173.244	172.5837	173.2402	176.9472	174.109	172.4483		176.9475		174.1255	173.1988		
C	89.1309	88.7181	89.057	86.4629	92.025	88.4833		86.4638		91.0099	89.4526		
H	29.5609	29.0043	29.6997	29.542	29.2584	28.9701		29.5422		29.5349	29.76		
H	29.849	29.9841	30.0722	29.9996	29.636	30.047		29.9997		29.9649	30.1125		
H	26.903	27.0325	26.8951	26.9982	26.9789	26.9628		26.9985		27.0665	26.8978		
H	29.3942	29.3194	29.3975	30.0361	29.3676	29.3056		30.0358		29.5864	29.4107		
H	29.1545	29.4205	29.1824	29.9393	29.9887	29.4052		29.9394		30.282	29.2006		
H	30.0925	30.379	29.8258	30.4837	30.5561	30.7386		30.4838		30.4112	29.8249		
H	30.9896	30.431	30.7738	30.1936	30.596	30.8771		30.1936		30.1505	30.8022		
H	30.6225	30.0631	30.4777	30.1513	30.2798	30.1997		30.1513		30.1944	30.5257		
H	27.2973	27.4024	27.5517	27.116	27.7061	27.837		27.1163		27.1305	27.606		
H	24.2101	23.6341	24.5327	23.541	24.388	24.1282		23.5408		23.7416	24.2096		
H	24.5211	24.6659	25.4251	24.5397	25.4762	25.0307		24.5397		24.5389	24.9777		
H	29.7254	29.7317	29.7277	29.1015	28.9755	29.7103		29.1015		29.7325	29.7158		
H	29.1295	29.1455	29.0771	29.0582	28.9923	29.1401		29.0583		29.1348	29.138		
H	29.1128	29.1198	28.9976	29.6793	29.6991	29.1935		29.6794		29.1007	29.2342		
H	28.2883	29.7472	28.3819	29.8302	29.2216	29.5532		29.8306		29.8163	28.4182		
H	29.3927	29.4619	29.3939	29.2006	29.8465	29.2941		29.2013		29.9105	29.3933		
H	29.8038	29.8496	29.8147	29.4189	29.813	29.7808		29.419		29.8658	29.8011		
H	29.771	29.8092	29.755	29.807	29.6671	29.765		29.807		29.7096	29.7636		
H	26.3845	26.2536	26.3602	26.4529	26.4129	26.2638		26.4528		26.3806	26.3763		
H	26.375	26.3429	26.3655	26.3503	26.0532	26.3482		26.3503		26.0314	26.3829		

Table S 7. Cartesian coordinates of the energy-minimized conformer 5*S*6*S*9*S*-2 optimized at the B3LYP/6-31G* level.

Atom	X	Y	Z
C	0.99320	1.50980	-0.07320
C	0.50370	0.05910	0.11260
O	1.60430	-0.77910	-0.34380
C	2.72910	0.01900	-0.78940
C	2.11270	1.38400	-1.11670
C	3.82950	-0.03050	0.26420
C	0.18970	-0.31250	1.55980
C	-0.67790	-0.29870	-0.85060
C	-1.98830	0.28580	-0.42740
C	-3.10170	-0.41350	-0.17270
C	-4.35990	0.27510	0.22870
C	-5.56250	-0.61780	0.47970
O	-4.42960	1.49010	0.35140
O	-0.78740	-1.70310	-0.97030
C	4.26210	-1.42720	0.63890
C	4.39530	1.05160	0.80700
H	1.39050	1.89320	0.87200
H	0.19140	2.18310	-0.39250
H	3.10720	-0.47630	-1.69430
H	1.69260	1.35130	-2.12790
H	2.82820	2.20860	-1.09020
H	-0.59780	0.33110	1.96620
H	-0.15260	-1.34980	1.61910
H	1.08790	-0.19950	2.17440
H	-0.42120	0.14520	-1.83230
H	-2.03940	1.37030	-0.33740
H	-3.10240	-1.49590	-0.26270
H	-5.34030	-1.34510	1.27120
H	-6.42310	-0.01170	0.76890
H	-5.80390	-1.19520	-0.42210
H	0.13310	-2.01360	-1.04620
H	3.41270	-2.00600	1.01710
H	4.64500	-1.96720	-0.23850
H	5.04760	-1.41420	1.40020
H	5.18980	0.95790	1.54310
H	4.10620	2.06570	0.54920

Table S 8. Cartesian coordinates of the energy-minimized conformer 5*S*6*S*9*S*-4 optimized at the B3LYP/6-31G* level.

Atom	X	Y	Z
C	-1.68840	-2.04030	-0.06080
C	-0.54310	-1.11050	0.39180
O	-0.94610	0.21100	-0.05220
C	-2.31580	0.22860	-0.49350
C	-2.91210	-1.11890	-0.03570
C	-3.05300	1.44990	0.01080
C	-0.37360	-1.09960	1.91300
C	0.78150	-1.42840	-0.37580
C	1.99620	-0.73280	0.18170
C	2.82110	0.05400	-0.51990
C	4.01250	0.68150	0.11780
C	4.87850	1.54510	-0.78300
O	4.28530	0.51590	1.29810
O	0.58930	-1.16440	-1.75470
C	-4.44390	1.62580	-0.54840
C	-2.50580	2.30530	0.87710
H	-1.79200	-2.91350	0.59100
H	-1.49380	-2.38830	-1.08030
H	-2.31600	0.25540	-1.59730
H	-3.72000	-1.46020	-0.68940
H	-3.31600	-1.02180	0.97850
H	0.09630	-2.02660	2.26420
H	0.24390	-0.25500	2.22960
H	-1.34710	-1.00650	2.40520
H	0.94930	-2.51240	-0.28960
H	2.23880	-0.90630	1.22850
H	2.64250	0.23610	-1.57640
H	4.28810	2.36800	-1.20640
H	5.71800	1.95060	-0.21510
H	5.25540	0.95660	-1.62940
H	0.18490	-0.27670	-1.76800
H	-4.42950	1.66800	-1.64650
H	-5.10090	0.78880	-0.27560
H	-4.90410	2.54740	-0.18080
H	-3.05390	3.17510	1.23010
H	-1.49710	2.16370	1.24810

Table S 9. Cartesian coordinates of the energy-minimized conformer 5*S*6*S*9*S*-6 optimized at the B3LYP/6-31G* level.

Atom	X	Y	Z
C	0.69860	1.64770	0.00380
C	0.37990	0.16560	0.31990
O	1.61390	-0.55990	0.06340
C	2.59440	0.30040	-0.54700
C	2.23410	1.69700	-0.01430
C	3.98440	-0.19980	-0.22120
C	0.00710	-0.08260	1.78050
C	-0.66310	-0.45280	-0.66290
C	-2.04700	0.09780	-0.48480
C	-3.12910	-0.63480	-0.18620
C	-4.50220	-0.09730	-0.01860
C	-4.75300	1.39440	-0.20020
O	-5.42010	-0.85470	0.26290
O	-0.67620	-1.85820	-0.53030
C	4.28270	-0.53640	1.21800
C	4.89340	-0.31840	-1.19380
H	0.25920	2.32740	0.73970
H	0.30400	1.92630	-0.98170
H	2.46080	0.28980	-1.64010
H	2.63670	2.49630	-0.64340
H	2.63000	1.83070	0.99800
H	-0.93240	0.41790	2.03570
H	-0.11930	-1.15380	1.95930
H	0.79640	0.29160	2.44030
H	-0.32330	-0.17110	-1.67900
H	-2.13390	1.17400	-0.62910
H	-3.03970	-1.70880	-0.05330
H	-4.47860	1.72070	-1.21050
H	-5.81310	1.59280	-0.03360
H	-4.15940	1.98500	0.50790
H	0.26320	-2.11370	-0.50600
H	4.13790	0.32860	1.87840
H	3.60810	-1.32150	1.57620
H	5.31460	-0.87960	1.33520
H	5.91220	-0.63640	-0.98840
H	4.65450	-0.09870	-2.23170

Table S 10. Cartesian coordinates of the energy-minimized conformer 5*R*6*S*9*S*-2 optimized at the B3LYP/6-31G* level.

Atom	X	Y	Z
C	0.37600	-0.45900	-1.12500
C	0.42080	0.66990	-0.07860
O	1.62360	0.40730	0.69450
C	2.43960	-0.60470	0.07730
C	1.85590	-0.79310	-1.33810
C	3.90800	-0.24140	0.11380
C	0.53530	2.06570	-0.69390
C	-0.74280	0.59070	0.96190
C	-2.09530	0.63090	0.32460
C	-3.03360	-0.31760	0.44310
C	-4.35410	-0.17180	-0.23120
C	-5.35100	-1.29480	-0.00380
O	-4.62790	0.79410	-0.92950
O	-0.60990	-0.57890	1.74490
C	4.84090	-1.32800	-0.36270
C	4.33820	0.94730	0.54240
H	-0.13660	-0.15620	-2.04250
H	-0.15360	-1.32040	-0.70470
H	2.30080	-1.54330	0.64200
H	2.01730	-1.80390	-1.72400
H	2.32630	-0.08710	-2.03220
H	-0.37190	2.33750	-1.24440
H	0.70490	2.81440	0.08730
H	1.37990	2.11020	-1.38870
H	-0.64450	1.48760	1.60230
H	-2.33550	1.51020	-0.27080
H	-2.84800	-1.20350	1.04340
H	-5.56790	-1.40080	1.06700
H	-6.27530	-1.08830	-0.54650
H	-4.93210	-2.25260	-0.33840
H	0.32830	-0.58270	2.00600
H	4.70550	-2.25180	0.21680
H	4.65850	-1.58800	-1.41420
H	5.88640	-1.02060	-0.26930
H	5.39770	1.18980	0.55680
H	3.64600	1.70290	0.89610

Table S 11. Cartesian coordinates of the energy-minimized conformer 5*R*6*S*9*S*-6 optimized at the B3LYP/6-31G* level.

Atom	X	Y	Z
C	-0.34230	0.13060	1.25090
C	-0.41260	0.63730	-0.20400
O	-1.65090	0.08920	-0.73340
C	-2.39650	-0.60970	0.29330
C	-1.81470	-0.08940	1.61740
C	-3.88040	-0.39990	0.08660
C	-0.48380	2.16140	-0.31360
C	0.71270	0.04630	-1.11640
C	2.09350	0.31900	-0.59720
C	2.98140	-0.62710	-0.25930
C	4.34970	-0.36680	0.25360
C	4.83640	1.06870	0.40320
O	5.07380	-1.30610	0.55160
O	0.52310	-1.34420	-1.27080
C	-4.36200	1.01230	-0.13000
C	-4.70920	-1.44820	0.11000
H	0.16780	0.83760	1.91190
H	0.20240	-0.81860	1.28140
H	-2.17610	-1.68440	0.21160
H	-1.95920	-0.80440	2.43250
H	-2.29400	0.85200	1.90850
H	0.45660	2.63140	-0.00410
H	-0.69840	2.46070	-1.34500
H	-1.28180	2.55570	0.32370
H	0.61470	0.55300	-2.09500
H	2.35740	1.37170	-0.51580
H	2.72360	-1.67740	-0.35640
H	4.19660	1.63410	1.09130
H	5.85610	1.05280	0.79190
H	4.82490	1.59200	-0.56040
H	-0.41810	-1.43160	-1.50370
H	-4.09940	1.66750	0.71090
H	-3.89620	1.44250	-1.02330
H	-5.44860	1.04280	-0.25240
H	-5.78460	-1.32980	0.00650
H	-4.34400	-2.46450	0.23860

Table S 12. Cartesian coordinates of the energy-minimized conformer 5*S*6*R*9*S*-1 optimized at the B3LYP/6-31G* level.

Atom	X	Y	Z
C	-1.47220	-1.04620	1.61460
C	-1.28770	-1.44750	0.12860
O	-1.52700	-0.23650	-0.63410
C	-2.04300	0.80360	0.21910
C	-1.43900	0.48920	1.59830
C	-1.72340	2.16470	-0.35800
C	-2.29480	-2.51380	-0.31300
C	0.15620	-1.89730	-0.26220
C	1.24940	-1.04810	0.33240
C	2.25280	-0.49350	-0.35940
C	3.29190	0.32650	0.32400
C	4.45120	0.79770	-0.53600
O	3.21950	0.61160	1.51150
O	0.24930	-1.97520	-1.67660
C	-0.29660	2.44700	-0.75590
C	-2.69510	3.07420	-0.48170
H	-0.71740	-1.48910	2.27170
H	-2.44800	-1.39890	1.96690
H	-3.13750	0.69810	0.27940
H	-2.01510	0.94080	2.41140
H	-0.41230	0.86320	1.66160
H	-2.13330	-2.78480	-1.35920
H	-2.19340	-3.41820	0.29930
H	-3.31580	-2.13500	-0.19870
H	0.29120	-2.92440	0.10890
H	1.24540	-0.89620	1.41050
H	2.33100	-0.64860	-1.43220
H	4.97490	-0.06110	-0.97520
H	4.08400	1.40630	-1.37250
H	5.14770	1.38430	0.06590
H	-0.15000	-1.14620	-1.99830
H	0.39640	2.32430	0.08540
H	0.03300	1.75080	-1.53480
H	-0.19260	3.46770	-1.13590
H	-2.49590	4.07700	-0.85070
H	-3.72560	2.85230	-0.21430

Table S 13. Cartesian coordinates of the energy-minimized conformer 5*S*6*R*9*S*-2 optimized at the B3LYP/6-31G* level.

Atom	X	Y	Z
C	0.52090	0.58450	1.30010
C	0.50320	1.08880	-0.15380
O	1.82310	0.77470	-0.66960
C	2.76070	0.47510	0.40240
C	1.99120	0.74890	1.70670
C	3.30250	-0.92740	0.16100
C	0.28400	2.59950	-0.27100
C	-0.47520	0.28560	-1.06810
C	-1.90430	0.41340	-0.64130
C	-2.69480	-0.60440	-0.27580
C	-4.10980	-0.36540	0.12300
C	-4.92000	-1.59670	0.49010
O	-4.60010	0.75460	0.15600
O	-0.10630	-1.07740	-1.10410
C	4.14970	-1.04970	-1.08320
C	3.04620	-1.97550	0.94950
H	0.22850	-0.46960	1.31810
H	-0.16610	1.14320	1.94190
H	3.59560	1.18100	0.29290
H	2.18620	1.77680	2.03090
H	2.29180	0.09340	2.52710
H	0.39130	2.92180	-1.31230
H	-0.70900	2.89290	0.08530
H	1.02790	3.14030	0.32340
H	-0.38210	0.73240	-2.07600
H	-2.33520	1.41290	-0.64640
H	-2.31610	-1.62220	-0.27640
H	-4.95590	-2.29560	-0.35560
H	-4.44680	-2.13200	1.32340
H	-5.93450	-1.30590	0.76920
H	0.86430	-1.07520	-1.18080
H	3.60430	-0.68920	-1.96360
H	5.05370	-0.43030	-1.00080
H	4.45990	-2.08320	-1.26360
H	3.44140	-2.96160	0.71840
H	2.44100	-1.90490	1.84790

Table S 14. Cartesian coordinates of the energy-minimized conformer 5*S*6*R*9*S*-6 optimized at the B3LYP/6-31G* level.

Atom	X	Y	Z
C	0.43680	0.54610	1.41710
C	0.40890	1.09970	-0.03100
O	1.72340	0.82080	-0.57920
C	2.60580	0.32130	0.43070
C	1.67880	-0.35300	1.46060
C	3.66210	-0.58050	-0.16740
C	0.16590	2.60810	-0.09090
C	-0.56630	0.31720	-0.96910
C	-1.99930	0.42050	-0.55480
C	-2.79660	-0.61560	-0.26160
C	-4.21780	-0.39750	0.12930
C	-5.03530	-1.64620	0.41040
O	-4.70620	0.71990	0.22020
O	-0.18680	-1.04400	-1.04960
C	4.64770	-1.15340	0.82150
C	3.72460	-0.83570	-1.47770
H	-0.48180	0.00770	1.66290
H	0.53690	1.37520	2.12710
H	3.11290	1.17610	0.91420
H	2.12990	-0.41520	2.45540
H	1.43040	-1.36520	1.12600
H	0.16070	2.96620	-1.12610
H	-0.78730	2.87850	0.37630
H	0.96450	3.13290	0.44350
H	-0.47050	0.79600	-1.96190
H	-2.42770	1.42000	-0.50380
H	-2.42010	-1.63280	-0.31820
H	-5.06080	-2.29270	-0.47630
H	-4.57490	-2.23220	1.21640
H	-6.05300	-1.36990	0.69230
H	0.76380	-1.02940	-1.25770
H	5.14560	-0.35730	1.39210
H	4.15470	-1.80480	1.55500
H	5.42010	-1.73920	0.31500
H	4.49090	-1.48910	-1.88660
H	3.03130	-0.38440	-2.17890

Table S 15. Cartesian coordinates of the energy-minimized conformer 5*S*6*R*9*S*-9 optimized at the B3LYP/6-31G* level.

Atom	X	Y	Z
C	0.41030	0.69060	1.30360
C	0.39440	0.92910	-0.23320
O	1.70460	0.51890	-0.70480
C	2.61840	0.45440	0.40550
C	1.74010	-0.03270	1.57040
C	3.80230	-0.41810	0.05280
C	0.17610	2.39720	-0.60600
C	-0.58880	-0.01770	-0.98950
C	-2.02710	0.19820	-0.62400
C	-2.82550	-0.73500	-0.08590
C	-4.25250	-0.53290	0.27000
C	-4.91090	0.81410	0.00200
O	-4.88620	-1.44890	0.77460
O	-0.23300	-1.36610	-0.76000
C	3.52310	-1.73860	-0.62070
C	5.04020	-0.01760	0.35750
H	-0.45310	0.10520	1.62830
H	0.37920	1.65090	1.83050
H	2.98190	1.46910	0.63130
H	2.17890	0.20500	2.54400
H	1.60160	-1.11700	1.51300
H	0.17320	2.53180	-1.69320
H	-0.76950	2.77680	-0.20300
H	0.98340	3.01110	-0.19310
H	-0.47660	0.22740	-2.06250
H	-2.41500	1.19110	-0.84270
H	-2.44250	-1.73070	0.11640
H	-4.39750	1.62000	0.54000
H	-4.88180	1.06190	-1.06580
H	-5.95000	0.76660	0.33220
H	0.72110	-1.40040	-0.94390
H	2.81330	-2.35050	-0.04860
H	3.08840	-1.58060	-1.61530
H	4.44130	-2.32060	-0.74210
H	5.90750	-0.64360	0.16480
H	5.23290	0.94970	0.81540

Table S 16. Cartesian coordinates of the energy-minimized conformer 5*S*6*R*9*S*-11 optimized at the B3LYP/6-31G* level.

Atom	X	Y	Z
C	0.60620	-1.53650	-0.40880
C	0.29930	-0.43990	0.64460
O	1.58170	0.16640	0.95250
C	2.65370	-0.52570	0.30390
C	2.00690	-1.18930	-0.92710
C	3.80010	0.41160	-0.00340
C	-0.29660	-1.00270	1.93390
C	-0.55560	0.74690	0.09060
C	-1.90580	0.33470	-0.41320
C	-3.06190	0.50160	0.24280
C	-4.35690	0.03890	-0.33550
C	-5.60400	0.35080	0.47320
O	-4.41600	-0.55500	-1.40140
O	0.12010	1.37560	-0.99000
C	4.98300	-0.22860	-0.68800
C	3.77090	1.70690	0.32400
H	-0.13800	-1.56980	-1.20790
H	0.60960	-2.51920	0.07720
H	3.02480	-1.31670	0.98090
H	2.56450	-2.06390	-1.27510
H	1.94220	-0.46690	-1.74650
H	-0.44990	-0.20890	2.67300
H	-1.25880	-1.48870	1.74180
H	0.38340	-1.74260	2.36850
H	-0.67700	1.45280	0.92880
H	-1.93900	-0.13250	-1.39620
H	-3.08850	0.99520	1.21340
H	-5.53530	-0.09680	1.47310
H	-5.70590	1.43450	0.61600
H	-6.48600	-0.03480	-0.04120
H	1.01760	1.55520	-0.65670
H	5.36610	-1.07840	-0.10640
H	4.71570	-0.62000	-1.67830
H	5.79960	0.48740	-0.81730
H	4.60500	2.36390	0.09150
H	2.92820	2.14160	0.85040

Table S 17. Cartesian coordinates of the energy-minimized conformer 5*R*6*R*9*S*-1 optimized at the B3LYP/6-31G* level.

Atom	X	Y	Z
C	1.03350	1.61380	0.59970
C	0.55340	0.72970	-0.57480
O	1.76170	0.15320	-1.15010
C	2.91170	0.44410	-0.32460
C	2.53930	1.77670	0.33560
C	3.24040	-0.73210	0.58960
C	-0.13270	1.51770	-1.68980
C	-0.30160	-0.48940	-0.10770
C	-1.65260	-0.10200	0.40240
C	-2.82140	-0.53970	-0.08360
C	-4.11120	-0.09590	0.51440
C	-5.37190	-0.67350	-0.10530
O	-4.16180	0.68860	1.45150
O	-0.42230	-1.42650	-1.16340
C	3.32250	-2.06970	-0.10770
C	3.48480	-0.61140	1.89840
H	0.87040	1.10920	1.55860
H	0.50470	2.57050	0.63260
H	3.74720	0.56250	-1.02800
H	2.71360	2.58340	-0.38450
H	3.11680	2.00850	1.23310
H	-0.37140	0.85730	-2.52810
H	-1.06470	1.96770	-1.33300
H	0.52800	2.31270	-2.05020
H	0.25470	-0.94090	0.73460
H	-1.67720	0.58220	1.25010
H	-2.84290	-1.23330	-0.91920
H	-6.25250	-0.27980	0.40560
H	-5.42210	-0.42440	-1.17320
H	-5.36370	-1.76890	-0.03570
H	0.45860	-1.44650	-1.57760
H	2.34290	-2.37880	-0.48980
H	3.99680	-2.02360	-0.97350
H	3.68510	-2.84920	0.56860
H	3.75290	-1.47620	2.50020
H	3.43690	0.33600	2.42580

Table S 18. Cartesian coordinates of the energy-minimized conformer 5*R*6*R*9*S*-2 optimized at the B3LYP/6-31G* level.

Atom	X	Y	Z
C	-2.06220	-1.60720	0.70400
C	-0.72690	-1.33280	-0.01860
O	-0.95590	-0.11860	-0.77390
C	-2.37180	0.19860	-0.86910
C	-3.10250	-1.00530	-0.24990
C	-2.57750	1.57190	-0.24420
C	-0.34980	-2.46270	-0.98280
C	0.41080	-0.99460	0.99510
C	1.75940	-0.81260	0.34810
C	2.53510	0.27000	0.48400
C	3.86710	0.34970	-0.17740
C	4.65750	1.62550	0.05870
O	4.30890	-0.55480	-0.87170
O	0.03460	0.11740	1.78740
C	-1.91060	2.69360	-1.00430
C	-3.25720	1.78700	0.88630
H	-2.06980	-1.08690	1.66550
H	-2.21830	-2.67530	0.88540
H	-2.60220	0.27910	-1.94040
H	-3.35510	-1.71810	-1.04240
H	-4.03780	-0.73230	0.24390
H	0.42880	-2.13800	-1.67770
H	0.01030	-3.34430	-0.43830
H	-1.22010	-2.76660	-1.57440
H	0.48470	-1.84950	1.68460
H	2.14610	-1.63470	-0.25150
H	2.20810	1.10620	1.09620
H	5.61130	1.57600	-0.46980
H	4.08860	2.49790	-0.28830
H	4.83740	1.77060	1.13180
H	-0.34050	0.76310	1.16060
H	-0.84310	2.48620	-1.14490
H	-2.34430	2.79460	-2.00900
H	-2.01680	3.65300	-0.48930
H	-3.35450	2.78710	1.30140
H	-3.74230	0.99210	1.44420

Table S 19. Cartesian coordinates of the energy-minimized conformer 5*R*6*R*9*S*-3 optimized at the B3LYP/6-31G* level.

Atom	X	Y	Z
C	0.62720	-0.06380	-1.61380
C	0.52720	-0.98650	-0.37710
O	1.88960	-1.12750	0.11860
C	2.77800	-0.21090	-0.56100
C	2.12930	-0.05960	-1.94180
C	2.99610	1.05410	0.26280
C	0.03760	-2.39620	-0.70940
C	-0.27000	-0.34230	0.80240
C	-1.75380	-0.30770	0.57820
C	-2.44020	0.77610	0.18820
C	-3.90150	0.82480	-0.07300
C	-4.74140	-0.42510	0.14350
O	-4.41090	1.86850	-0.45620
O	-0.03050	-1.08380	1.99280
C	3.37280	0.81160	1.70580
C	2.91120	2.29070	-0.23780
H	0.28600	0.94800	-1.37130
H	0.01560	-0.42750	-2.44410
H	3.73950	-0.73850	-0.62210
H	2.38940	-0.93540	-2.54630
H	2.45240	0.82590	-2.49340
H	0.05160	-3.02510	0.18530
H	-0.98450	-2.37410	-1.10260
H	0.68820	-2.85040	-1.46350
H	0.10590	0.68700	0.90990
H	-2.26450	-1.25240	0.75530
H	-1.93770	1.73000	0.03660
H	-5.78300	-0.19000	-0.08190
H	-4.40660	-1.24280	-0.50580
H	-4.66340	-0.77740	1.17880
H	0.91870	-1.30190	1.95980
H	2.54480	0.35700	2.26150
H	4.22100	0.11810	1.78220
H	3.64600	1.74460	2.20710
H	3.11430	3.16070	0.38160
H	2.64610	2.49600	-1.27010

Table S 20. Cartesian coordinates of the energy-minimized conformer 5*R*6*R*9*S*-4 optimized at the B3LYP/6-31G* level.

Atom	X	Y	Z
C	0.76600	1.20880	1.45530
C	0.87730	1.55520	-0.05340
O	1.61250	0.47490	-0.66220
C	2.22350	-0.33620	0.35890
C	1.23840	-0.25110	1.53820
C	2.50950	-1.72440	-0.17080
C	1.63680	2.86530	-0.29620
C	-0.51190	1.60260	-0.77170
C	-1.27830	0.33190	-0.59750
C	-2.51660	0.21890	-0.10000
C	-3.14750	-1.12190	0.05020
C	-4.59810	-1.13720	0.49900
O	-2.53750	-2.16030	-0.16820
O	-1.29780	2.69350	-0.30490
C	1.42700	-2.42040	-0.95690
C	3.69060	-2.29640	0.08420
H	-0.25110	1.35860	1.82680
H	1.42820	1.85920	2.03840
H	3.17700	0.12620	0.66120
H	1.71740	-0.49730	2.49070
H	0.40390	-0.94370	1.38950
H	1.71080	3.08370	-1.36830
H	1.15230	3.70900	0.20940
H	2.65430	2.78400	0.09920
H	-0.29180	1.71280	-1.84830
H	-0.76170	-0.57210	-0.90390
H	-3.08120	1.09870	0.19560
H	-4.70170	-0.63950	1.47190
H	-5.22350	-0.57970	-0.21010
H	-4.95480	-2.16610	0.57370
H	-0.91490	3.50950	-0.65830
H	0.48240	-2.49120	-0.40330
H	1.21130	-1.86830	-1.87920
H	1.73390	-3.43480	-1.22890
H	3.91430	-3.31130	-0.23430
H	4.47630	-1.77420	0.62540

Table S 21. Cartesian coordinates of the energy-minimized conformer 5*R*6*R*9*S*-5 optimized at the B3LYP/6-31G* level.

Atom	X	Y	Z
C	-1.55100	-2.10590	0.14470
C	-0.41010	-1.16750	-0.34320
O	-1.03260	0.12750	-0.54990
C	-2.45820	0.00840	-0.53440
C	-2.73350	-1.16740	0.42270
C	-3.11470	1.31370	-0.14730
C	0.21660	-1.64630	-1.65320
C	0.64890	-0.93690	0.76930
C	1.77030	-0.02260	0.34290
C	3.06560	-0.36650	0.32830
C	4.19370	0.51890	-0.05990
C	3.91000	1.96260	-0.44780
O	5.33410	0.07780	-0.06230
O	0.01490	-0.40630	1.93190
C	-4.62380	1.28980	-0.13960
C	-2.41020	2.40800	0.15380
H	-1.25880	-2.67190	1.03290
H	-1.80250	-2.82440	-0.64350
H	-2.80400	-0.26720	-1.54740
H	-3.70520	-1.63750	0.24480
H	-2.70480	-0.81090	1.45750
H	0.95810	-0.93350	-2.02370
H	0.71260	-2.61470	-1.51390
H	-0.55660	-1.76890	-2.41910
H	1.06220	-1.90750	1.06450
H	1.46290	0.98220	0.05560
H	3.37740	-1.36980	0.61540
H	3.23940	2.01440	-1.31380
H	3.42450	2.50180	0.37420
H	4.85440	2.45120	-0.69320
H	-0.51220	0.34800	1.61090
H	-5.02130	0.97140	-1.11310
H	-5.01490	0.58490	0.60580
H	-5.03290	2.27900	0.08490
H	-2.90430	3.33490	0.43310
H	-1.32680	2.41700	0.11310

Table S 22. Cartesian coordinates of the energy-minimized conformer 5*R*6*R*9*S*-6 optimized at the B3LYP/6-31G* level.

Atom	X	Y	Z
C	-1.71990	-1.84110	0.49900
C	-0.51590	-1.13160	-0.15290
O	-1.07360	0.05050	-0.78150
C	-2.52410	-0.01000	-0.85540
C	-2.89410	-1.43200	-0.40080
C	-3.07750	1.16980	-0.06710
C	0.16660	-1.99140	-1.22040
C	0.49330	-0.61210	0.90790
C	1.66200	0.11480	0.29620
C	2.94860	-0.22200	0.45690
C	4.04870	0.57800	-0.15320
C	5.46360	0.10490	0.13420
O	3.83150	1.56110	-0.84560
O	-0.16300	0.24700	1.83590
C	-2.76530	2.51440	-0.67950
C	-3.75630	1.05240	1.07840
H	-1.85840	-1.46300	1.51570
H	-1.57940	-2.92540	0.54770
H	-2.78830	0.13800	-1.91150
H	-2.94950	-2.08550	-1.27820
H	-3.86390	-1.48370	0.09910
H	0.88230	-1.39850	-1.79630
H	0.70400	-2.83030	-0.76150
H	-0.57380	-2.40160	-1.91570
H	0.85280	-1.46880	1.49030
H	1.43010	0.99800	-0.29770
H	3.22650	-1.09160	1.05180
H	6.18200	0.75900	-0.36310
H	5.65430	0.10330	1.21530
H	5.60180	-0.92680	-0.21470
H	-0.71820	0.84090	1.29800
H	-1.68680	2.62760	-0.84180
H	-3.24160	2.61290	-1.66490
H	-3.11200	3.33850	-0.04910
H	-4.10950	1.92990	1.61440
H	-3.99020	0.09360	1.53070

Table S 23. Cartesian coordinates of the energy-minimized conformer 5*R*6*R*9*S*-8 optimized at the B3LYP/6-31G* level.

Atom	X	Y	Z
C	0.76590	1.20880	1.45520
C	0.87710	1.55520	-0.05330
O	1.61240	0.47490	-0.66230
C	2.22370	-0.33590	0.35890
C	1.23870	-0.25100	1.53830
C	2.50990	-1.72410	-0.17080
C	1.63650	2.86540	-0.29610
C	-0.51210	1.60250	-0.77170
C	-1.27840	0.33180	-0.59750
C	-2.51680	0.21880	-0.10010
C	-3.14750	-1.12210	0.05020
C	-4.59810	-1.13730	0.49920
O	-2.53760	-2.16050	-0.16840
O	-1.29810	2.69340	-0.30510
C	1.42740	-2.42040	-0.95670
C	3.69110	-2.29600	0.08400
H	-0.25130	1.35820	1.82680
H	1.42770	1.85930	2.03850
H	3.17710	0.12660	0.66100
H	1.71790	-0.49690	2.49080
H	0.40440	-0.94390	1.38980
H	1.71060	3.08370	-1.36820
H	1.15180	3.70900	0.20940
H	2.65400	2.78420	0.09940
H	-0.29190	1.71260	-1.84830
H	-0.76180	-0.57220	-0.90370
H	-3.08140	1.09850	0.19540
H	-4.70170	-0.63950	1.47200
H	-5.22360	-0.58000	-0.21000
H	-4.95480	-2.16620	0.57410
H	-0.91530	3.50940	-0.65850
H	0.48280	-2.49110	-0.40300
H	1.21160	-1.86860	-1.87910
H	1.73430	-3.43480	-1.22850
H	3.91490	-3.31080	-0.23440
H	4.47680	-1.77360	0.62510

Table S 24. Cartesian coordinates of the energy-minimized conformer 5*R*6*R*9*S*-10 optimized at the B3LYP/6-31G* level.

Atom	X	Y	Z
C	0.74030	1.17700	1.48520
C	0.80430	1.60930	-0.00110
O	1.60830	0.62180	-0.67440
C	2.22170	-0.25950	0.27190
C	1.25800	-0.26880	1.47540
C	2.50620	-1.61220	-0.33960
C	1.46380	2.98150	-0.18270
C	-0.59130	1.58960	-0.70680
C	-1.29370	0.27520	-0.56750
C	-2.53990	0.11120	-0.10370
C	-3.14530	-1.24570	0.00250
C	-4.59070	-1.29960	0.46810
O	-2.52300	-2.26370	-0.26470
O	-1.43380	2.62440	-0.20970
C	3.21470	-2.58540	0.57080
C	2.16860	-1.91260	-1.59590
H	-0.27170	1.27290	1.88760
H	1.39700	1.81470	2.08830
H	3.18370	0.18020	0.59600
H	1.75410	-0.54850	2.40970
H	0.44590	-0.98040	1.29330
H	1.50480	3.26110	-1.24250
H	0.92790	3.75940	0.37320
H	2.49120	2.95150	0.19390
H	-0.38320	1.74000	-1.78100
H	-0.73220	-0.60280	-0.87710
H	-3.13430	0.96950	0.19630
H	-4.92930	-2.33640	0.51230
H	-4.69160	-0.83600	1.45800
H	-5.23400	-0.73030	-0.21530
H	-1.10600	3.46680	-0.55620
H	4.15510	-2.16370	0.95270
H	2.60370	-2.83840	1.44750
H	3.44990	-3.51590	0.04620
H	2.38200	-2.89220	-2.01570
H	1.67470	-1.19140	-2.23670

Table S 25. Cartesian coordinates of the energy-minimized conformer 5*R*6*R*9*S*-11 optimized at the B3LYP/6-31G* level.

Atom	X	Y	Z
C	-0.62550	0.13550	-1.61030
C	-0.57220	1.02180	-0.34450
O	-1.94220	1.08330	0.14830
C	-2.78380	0.14890	-0.56340
C	-2.12460	0.07170	-1.94550
C	-2.94630	-1.15110	0.21840
C	-0.14570	2.46190	-0.62860
C	0.24930	0.37560	0.81780
C	1.72970	0.41790	0.59040
C	2.48120	-0.62710	0.21730
C	3.94650	-0.48630	-0.02180
C	4.69830	-1.76210	-0.36030
O	4.51600	0.59200	0.05240
O	-0.02830	1.06490	2.03070
C	-3.31810	-0.97280	1.67190
C	-2.82260	-2.36580	-0.32610
H	-0.23870	-0.86680	-1.39750
H	-0.02840	0.55380	-2.42520
H	-3.76920	0.63200	-0.61250
H	-2.42300	0.95350	-2.52280
H	-2.40470	-0.80970	-2.52660
H	-0.18830	3.05910	0.28660
H	0.87690	2.49910	-1.01840
H	-0.81610	2.91100	-1.36840
H	-0.08200	-0.67260	0.89010
H	2.22010	1.37870	0.73690
H	2.04190	-1.61640	0.09540
H	5.75530	-1.53940	-0.51720
H	4.59310	-2.49450	0.45060
H	4.28150	-2.22560	-1.26390
H	-0.98450	1.24960	1.99530
H	-2.50160	-0.50850	2.23660
H	-4.18920	-0.31260	1.77900
H	-3.55430	-1.93180	2.14200
H	-2.98980	-3.26360	0.26390
H	-2.56130	-2.52520	-1.36750

ECD calculation

Table S 26. Energy and Boltzmann distribution of the energy-minimized seven conformers optimized at the B3LYP/6-31G* level with PCM in MeOH for ECD caluculation.

The optimized conformers which showed >1% Boltzmann population were shown.

Conformation No.	1	2	3	4	5	6	7
Energy (A.U.)	-733.0603	-733.0637	-733.0601	-733.0599	-733.0628	-733.0604	-733.0601
Energy (kcal/mol)	-460002.7	-460004.8	-460002.5	-460002.4	-460004.2	-460002.7	-460002.5
Relative Energy (kcal/mol)	2.12	0.00	2.26	2.35	0.59	2.07	2.25
Boltzmann population (%)	1.87%	67.21%	1.49%	1.26%	24.63%	2.05%	1.50%

Table S 27. Cartesian coordinates of the energy-minimized conformer 1 optimized at the B3LYP/6-31G* level with PCM in MeOH of garcienone.

Atom	X	Y	Z
C	1.11350	-1.12960	1.67870
C	0.89510	-1.47140	0.18100
O	1.22240	-0.26260	-0.55480
C	1.39010	0.84800	0.35180
C	1.90540	0.18880	1.64090
C	2.29770	1.88280	-0.27650
C	1.85440	-2.56610	-0.29590
C	-0.57030	-1.83660	-0.23030
C	-1.62690	-0.90970	0.31500
C	-2.46960	-0.18190	-0.43160
C	-3.49930	0.69380	0.18850
C	-4.38980	1.45860	-0.76930
O	-3.62780	0.79660	1.40510
O	-0.63130	-1.92600	-1.65000
C	3.59780	1.40410	-0.87330
C	1.93760	3.17100	-0.27520
H	1.65670	-1.92860	2.19050
H	0.16990	-0.98880	2.21070
H	0.40930	1.30440	0.54830
H	1.73580	0.81710	2.51980
H	2.98000	-0.00680	1.56460
H	1.61990	-3.52070	0.18830
H	1.78170	-2.70070	-1.37790
H	2.88430	-2.29380	-0.04350
H	-0.78320	-2.84470	0.15270
H	-1.73600	-0.85070	1.39590
H	-2.42710	-0.22680	-1.51600
H	-4.93020	0.76130	-1.42200
H	-5.10390	2.07170	-0.21620
H	-3.78320	2.09740	-1.42330
H	-0.17520	-1.12430	-1.96750
H	4.22450	0.89610	-0.12930
H	3.41170	0.67910	-1.67340
H	4.17180	2.23930	-1.28510
H	2.58450	3.94520	-0.68050
H	0.98670	3.49880	0.13880

Table S 28. Cartesian coordinates of the energy-minimized conformer 2 optimized at the B3LYP/6-31G* level with PCM in MeOH of garcienone.

Atom	X	Y	Z
C	0.37450	-0.48910	-1.10970
C	0.41980	0.66610	-0.09290
O	1.62100	0.42040	0.68920
C	2.44080	-0.60540	0.09110
C	1.85440	-0.82720	-1.31760
C	3.90820	-0.23670	0.11480
C	0.53420	2.04520	-0.74450
C	-0.74320	0.61940	0.95210
C	-2.09690	0.64860	0.31730
C	-3.03190	-0.30440	0.44100
C	-4.35550	-0.17820	-0.22360
C	-5.32240	-1.32500	-0.01000
O	-4.65670	0.79590	-0.90770
O	-0.61270	-0.52740	1.77250
C	4.84090	-1.33530	-0.33460
C	4.34060	0.96430	0.50910
H	-0.13800	-0.20880	-2.03410
H	-0.15140	-1.34270	-0.66940
H	2.30720	-1.52850	0.67910
H	2.01450	-1.84720	-1.67710
H	2.32060	-0.13870	-2.03130
H	-0.37350	2.30180	-1.30100
H	0.70380	2.81510	0.01580
H	1.37600	2.06800	-1.44340
H	-0.64240	1.53390	1.56520
H	-2.33470	1.52740	-0.27900
H	-2.84140	-1.19100	1.03770
H	-5.52700	-1.45270	1.06050
H	-6.25690	-1.13750	-0.54230
H	-4.87960	-2.26550	-0.36110
H	0.33490	-0.54720	2.00060
H	4.70410	-2.24280	0.26920
H	4.65250	-1.62250	-1.37750
H	5.88650	-1.02500	-0.25270
H	5.40050	1.20620	0.51310
H	3.65080	1.73280	0.84020

Table S 29. Cartesian coordinates of the energy-minimized conformer 3 optimized at the B3LYP/6-31G* level with PCM in MeOH of garcienone.

Atom	X	Y	Z
C	-0.50350	0.57880	1.72520
C	-0.55930	1.34360	0.38470
O	-1.28000	0.46990	-0.52920
C	-1.71060	-0.73310	0.12790
C	-1.69370	-0.38530	1.62860
C	-3.04690	-1.20720	-0.39940
C	-1.34170	2.65610	0.49650
C	0.82130	1.59770	-0.27890
C	1.69260	0.37030	-0.36700
C	2.92830	0.28000	0.14780
C	3.82210	-0.89670	0.03420
C	3.35630	-2.12040	-0.73330
O	4.92990	-0.86630	0.56410
O	0.62440	2.10530	-1.60530
C	-3.47020	-2.57230	0.08590
C	-3.79020	-0.47190	-1.23110
H	-0.57040	1.25520	2.58190
H	0.43140	0.01650	1.81550
H	-0.96400	-1.52390	-0.06010
H	-1.58010	-1.26830	2.26330
H	-2.63000	0.11460	1.90130
H	-0.80990	3.36890	1.13690
H	-1.47280	3.10900	-0.48960
H	-2.32950	2.47160	0.93040
H	1.34620	2.38300	0.27520
H	1.26900	-0.45920	-0.93150
H	3.35680	1.11110	0.70620
H	3.12810	-1.86530	-1.77450
H	4.14300	-2.87670	-0.71090
H	2.44280	-2.53500	-0.29190
H	-0.04500	1.51850	-2.00330
H	-2.72090	-3.33380	-0.16950
H	-3.58000	-2.59590	1.17820
H	-4.42510	-2.87080	-0.35630
H	-4.75070	-0.83060	-1.59270
H	-3.45940	0.50260	-1.57340

Table S 30. Cartesian coordinates of the energy-minimized conformer 4 optimized at the B3LYP/6-31G* level with PCM in MeOH of garcienone.

Atom	X	Y	Z
C	-0.55850	-0.36150	1.10130
C	-0.56830	0.45660	-0.21690
O	-1.68550	-0.06440	-0.99150
C	-2.53750	-0.89300	-0.15730
C	-1.56260	-1.49920	0.85770
C	-3.71000	-0.07580	0.37250
C	-0.76480	1.95650	0.01100
C	0.66900	0.17380	-1.13140
C	1.97320	0.54800	-0.50460
C	2.99590	-0.29000	-0.28080
C	4.26270	0.18530	0.33510
C	5.34030	-0.86000	0.53910
O	4.43470	1.35620	0.66220
O	0.68800	-1.18910	-1.52050
C	-4.53590	0.59180	-0.70100
C	-4.02200	0.02840	1.66870
H	-0.88230	0.27180	1.93300
H	0.44080	-0.73510	1.33820
H	-2.94210	-1.65710	-0.83380
H	-1.05750	-2.34980	0.39040
H	-2.04340	-1.85960	1.76960
H	0.04190	2.37340	0.62370
H	-0.79420	2.49990	-0.93990
H	-1.70880	2.12770	0.53620
H	0.53560	0.81610	-2.02160
H	2.09630	1.59340	-0.22860
H	2.92150	-1.33820	-0.55350
H	5.61330	-1.31460	-0.42160
H	6.22360	-0.41010	0.99660
H	4.96640	-1.67070	1.17700
H	-0.22740	-1.36580	-1.80380
H	-3.91810	1.26570	-1.30470
H	-4.95350	-0.15390	-1.39190
H	-5.36440	1.16410	-0.27350
H	-4.88490	0.60610	1.99170
H	-3.44830	-0.45090	2.45580

Table S 31. Cartesian coordinates of the energy-minimized conformer 5 optimized at the B3LYP/6-31G* level with PCM in MeOH of garcienone.

Atom	X	Y	Z
C	-0.34450	0.14900	1.24740
C	-0.41270	0.61420	-0.22060
O	-1.64450	0.03740	-0.73830
C	-2.40560	-0.60930	0.31620
C	-1.81820	-0.04850	1.62090
C	-3.88560	-0.38250	0.09500
C	-0.49040	2.13390	-0.37240
C	0.71810	0.00690	-1.11690
C	2.09400	0.30170	-0.60190
C	2.99070	-0.62930	-0.24090
C	4.35270	-0.34310	0.26180
C	4.82280	1.09530	0.38980
O	5.09120	-1.27560	0.57120
O	0.53500	-1.39050	-1.24600
C	-4.35410	1.03340	-0.12950
C	-4.72550	-1.42340	0.11420
H	0.17060	0.87210	1.88600
H	0.19120	-0.80420	1.30710
H	-2.20360	-1.68880	0.27240
H	-1.96500	-0.73680	2.45790
H	-2.28740	0.90590	1.88260
H	0.44590	2.61530	-0.07030
H	-0.69900	2.40480	-1.41290
H	-1.29270	2.53980	0.25170
H	0.62270	0.49210	-2.10550
H	2.34890	1.35740	-0.54520
H	2.74190	-1.68420	-0.31050
H	4.17500	1.66100	1.06920
H	5.84370	1.10360	0.77610
H	4.80000	1.60310	-0.58120
H	-0.41630	-1.48610	-1.43580
H	-4.10560	1.68430	0.71860
H	-3.86690	1.46560	-1.01070
H	-5.43740	1.07020	-0.27630
H	-5.79880	-1.29390	-0.00130
H	-4.37080	-2.44200	0.25420

Table S 32. Cartesian coordinates of the energy-minimized conformer 6 optimized at the B3LYP/6-31G* level with PCM in MeOH of garcienone.

Atom	X	Y	Z
C	1.57740	2.02010	-0.00560
C	0.50320	0.96000	0.33740
O	1.10810	-0.32050	0.03800
C	2.43390	-0.15620	-0.48610
C	2.88610	1.22170	0.03040
C	3.33100	-1.30890	-0.09330
C	0.12420	0.96830	1.82110
C	-0.74190	1.09620	-0.59200
C	-1.73120	-0.01850	-0.39750
C	-3.05900	0.13190	-0.28840
C	-3.96320	-1.03460	-0.10590
C	-5.44370	-0.72480	-0.01630
O	-3.54260	-2.18520	-0.03120
O	-1.32180	2.39330	-0.50860
C	4.68500	-1.31410	-0.76050
C	2.95000	-2.24850	0.77690
H	1.56780	2.85850	0.69590
H	1.40480	2.42630	-1.00820
H	2.37950	-0.12030	-1.58850
H	3.67490	1.66590	-0.58280
H	3.26210	1.12350	1.05530
H	-0.41740	1.88290	2.09010
H	-0.50510	0.10750	2.06870
H	1.02590	0.92280	2.44010
H	-0.35830	1.02130	-1.61900
H	-1.31120	-1.02100	-0.35380
H	-3.51420	1.11730	-0.34710
H	-5.77880	-0.20790	-0.92440
H	-6.01650	-1.64490	0.11480
H	-5.63870	-0.04620	0.82370
H	-1.73630	2.48030	0.36590
H	4.58790	-1.32130	-1.85460
H	5.26420	-0.41710	-0.50410
H	5.26910	-2.19000	-0.46360
H	3.61400	-3.06530	1.04880
H	1.96770	-2.22870	1.23620

Table S 33. Cartesian coordinates of the energy-minimized conformer 7 optimized at the B3LYP/6-31G* level with PCM in MeOH of garcienone.

Atom	X	Y	Z
C	-1.58210	-2.06320	-0.09540
C	-0.50750	-1.01710	0.29390
O	-1.10300	0.27540	0.02770
C	-2.40170	0.12450	-0.58460
C	-2.88500	-1.25230	-0.10290
C	-3.27630	1.30420	-0.21800
C	-0.14680	-1.07130	1.78070
C	0.74860	-1.13190	-0.62420
C	1.73600	-0.01990	-0.38710
C	3.06190	-0.18550	-0.26800
C	4.03800	0.90360	-0.03880
C	3.55050	2.33570	0.08800
O	5.23420	0.63550	0.04620
O	1.32820	-2.42950	-0.56880
C	-3.36340	1.68380	1.23920
C	-3.94530	1.95930	-1.17320
H	-1.60670	-2.90500	0.60170
H	-1.37670	-2.46740	-1.09250
H	-2.28290	0.09820	-1.67840
H	-3.65100	-1.67210	-0.76110
H	-3.30600	-1.17970	0.90570
H	0.38760	-1.99590	2.02860
H	0.48260	-0.22120	2.06270
H	-1.05640	-1.04100	2.38900
H	0.37870	-1.02930	-1.65390
H	1.28990	0.96950	-0.32430
H	3.50460	-1.17560	-0.34960
H	2.85150	2.43890	0.92580
H	4.40810	2.99110	0.25100
H	3.02110	2.65120	-0.81830
H	1.72840	-2.54230	0.30940
H	-3.73800	0.85520	1.85370
H	-2.37280	1.94060	1.63070
H	-4.02910	2.53970	1.38370
H	-4.61370	2.78450	-0.93950
H	-3.85470	1.68900	-2.22290
